# Tumor LINE-1 methylation level and colorectal cancer location in relation to patient survival

**DOI:** 10.18632/oncotarget.10398

**Published:** 2016-07-04

**Authors:** Kosuke Mima, Jonathan A. Nowak, Zhi Rong Qian, Yin Cao, Mingyang Song, Yohei Masugi, Yan Shi, Annacarolina da Silva, Mancang Gu, Wanwan Li, Tsuyoshi Hamada, Xuehong Zhang, Kana Wu, Jeffrey A. Meyerhardt, Hideo Baba, Edward L. Giovannucci, Andrew T. Chan, Charles S. Fuchs, Shuji Ogino, Reiko Nishihara

**Affiliations:** ^1^ Department of Medical Oncology, Dana-Farber Cancer Institute and Harvard Medical School, Boston, MA, USA; ^2^ Division of MPE Molecular Pathological Epidemiology, Department of Pathology, Brigham and Women's Hospital and Harvard Medical School, Boston, MA, USA; ^3^ Clinical and Translational Epidemiology Unit, Massachusetts General Hospital and Harvard Medical School, Boston, MA, USA; ^4^ Division of Gastroenterology, Massachusetts General Hospital, Boston, MA, USA; ^5^ Department of Nutrition, Harvard T.H. Chan School of Public Health, Boston, MA, USA; ^6^ Channing Division of Network Medicine, Department of Medicine, Brigham and Women's Hospital and Harvard Medical School, Boston, MA, USA; ^7^ Department of Gastroenterological Surgery, Graduate School of Medical Science, Kumamoto University, Kumamoto, Japan; ^8^ Department of Epidemiology, Harvard T.H. Chan School of Public Health, Boston, MA, USA

**Keywords:** epigenetics, left-sided, molecular pathological epidemiology, prognosis, right-sided

## Abstract

Colorectal tumors arise with genomic and epigenomic alterations through interactions between neoplastic cells, immune cells, and microbiota that vary along the proximal to distal axis of colorectum. Long interspersed nucleotide element-1 (LINE-1) hypomethylation in colorectal cancer has been associated with worse clinical outcome. Utilizing 1,317 colon and rectal carcinoma cases in two U.S.-nationwide prospective cohort studies, we examined patient survival according to LINE-1 methylation level stratified by tumor location. Cox proportional hazards model was used to assess a statistical interaction between LINE-1 methylation level and tumor location in colorectal cancer-specific mortality analysis, controlling for potential confounders including microsatellite instability, CpG island methylator phenotype, and *KRAS*, *BRAF*, and *PIK3CA* mutations. A statistically significant interaction was found between LINE-1 methylation level and tumor location in colorectal cancer-specific mortality analysis (*P*_interaction_ = 0.011). The association of LINE-1 hypomethylation with higher colorectal cancer-specific mortality was stronger in proximal colon cancers (multivariable hazard ratio [HR], 1.66; 95% confidence interval [CI], 1.21 to 2.28) than in distal colon cancers (multivariable HR, 1.18; 95% CI, 0.81 to 1.72) or rectal cancers (multivariable HR, 0.87; 95% CI, 0.57 to 1.34). Our data suggest the interactive effect of LINE-1 methylation level and colorectal cancer location on clinical outcome.

## INTRODUCTION

Evidence suggests that colorectal tumors arise with sets of genomic and epigenomic alterations through interactions between neoplastic cells, immune cells, and microbiota that vary along the proximal to distal axis of colorectum [1–6]. Consistent with a continuous change in the intestinal microbiota and luminal contents along the bowel subsites, host immunity against colorectal tumors and proportions of colorectal cancers with specific molecular features of colorectal cancer such as microsatellite instability (MSI), high-level CpG island methylator phenotype (CIMP), and *BRAF* and *PIK3CA* mutations change along the bowel subsites [7–12].

Methylation status of the long interspersed nucleotide element-1 (LINE-1), which constitutes approximately 18% of the entire human genome, serves as a surrogate for overall cellular DNA methylation status [13, 14]. The genome-wide DNA hypomethylation has been associated with an increased chromosomal instability that may cause low-level antitumor immunity in colorectal cancer [15–20]. In fact, LINE-1 hypomethylation in colorectal cancer has been associated with a lower density of T cells in tumor tissue and worse clinical outcome [21–23]. Studies have shown that the prognostic association of tumor LINE-1 methylation level in colorectal cancer differs by MSI status [24, 25]. We hypothesized that the prognostic association of LINE-1 hypomethylation in colorectal cancer might differ by tumor location.

To test this hypothesis, we utilized resources of 1,317 colorectal cancer cases in two U.S.-nationwide prospective cohort studies (the Nurses’ Health Study [NHS] and the Health Professionals Follow-up Study [HPFS]), and examined the interactive association of LINE-1 methylation level and tumor location in colorectal cancer mortality analysis, controlling for potential confounders including major molecular features of colorectal cancer. A better understanding of the prognostic association of tumor LINE-1 hypomethylation according to colorectal cancer location may offer new insights into the pathogenesis of colorectal cancer.

## RESULTS

### Tumor LINE-1 methylation level and colorectal cancer location

We measured tumor LINE-1 methylation level (ranging from 23.1 to 93.8% of 0 to 100% scale; mean 63.4%; standard deviation 9.8%) among 1,317 colon and rectal cancer cases within the NHS and the HPFS. Table 1 summarizes clinical, pathological, and tumor molecular features according to tumor LINE-1 methylation level in colorectal cancer. Of the 1,317 cases, 621 (47%) had proximal colon cancer, 409 (31%) had distal colon cancer, and 287 (22%) had rectal cancer. Low-level tumor LINE-1 methylation was associated with higher pN stage and metastatic disease, and inversely associated with poor tumor differentiation, MSI-high, *MLH1* hypermethylation, CIMP-high, and *BRAF* mutation (*P* ≤ 0.003 with the adjusted α level of 0.003 for multiple hypothesis testing).

**Table 1:** Clinical, pathological, and tumor molecular features according to tumor LINE-1 methylation level in colorectal cancer

Characteristic^a^	Total No. (n = 1,317)	Tumor LINE-1 methylation level			*P* value^b^
		High (≥65%) (n = 579)	Intermediate (55-64.9%) (n = 496)	Low (<55%) (n = 242)	
Mean age ± SD (year)	68.9 ± 8.8	69.9 ± 8.6	68.7 ± 8.7	67.1 ± 9.1	0.0001
Sex					0.09
Men	593 (45%)	255 (44%)	214 (43%)	124 (51%)	
Women	724 (55%)	324 (56%)	282 (57%)	118 (49%)	
Year of diagnosis					< 0.0001
Prior to 1995	468 (36%)	162 (28%)	202 (40%)	104 (43%)	
1996 to 2000	402 (30%)	157 (27%)	147 (30%)	98 (40%)	
2001 to 2008	447 (34%)	260 (45%)	147 (30%)	40 (17%)	
Family history of colorectal cancer in a first-degree relative					0.35
Absent	1,048 (80%)	471 (81%)	391 (79%)	186 (77%)	
Present	264 (20%)	107 (19%)	102 (21%)	55 (23%)	
Tumor location					0.004
Proximal colon	621 (47%)	301 (52%)	224 (45%)	96 (40%)	
Distal colon	409 (31%)	151 (26%)	166 (34%)	92 (38%)	
Rectum	287 (22%)	127 (22%)	106 (21%)	54 (22%)	
Tumor differentiation					0.002
Well to moderate	1,178 (90%)	501 (87%)	461 (94%)	216 (90%)	
Poor	131 (10%)	74 (13%)	32 (6.5%)	25 (10%)	
pT stage (depth of tumour invasion)					0.45
pT1 (submucosa)	143 (12%)	62 (12%)	63 (14%)	18 (8.2%)	
pT2 (muscularis propria)	249 (21%)	115 (22%)	90 (19%)	44 (20%)	
pT3 (subserosa)	757 (62%)	327 (62%)	288 (62%)	142 (65%)	
pT4 (serosa or other organs)	64 (5.3%)	26 (4.9%)	23 (5.0%)	15 (6.9%)	
pN stage (number of positive lymph nodes)					0.003
pN0 (0)	740 (63%)	348 (68%)	280 (62%)	112 (54%)	
pN1 (1-3)	268 (23%)	108 (21%)	105 (23%)	55 (26%)	
pN2 (≥4)	163 (14%)	56 (11%)	65 (15%)	42 (20%)	
TNM stage^c^					0.0001
I	308 (26%)	141 (27%)	124 (27%)	43 (19%)	
II	385 (32%)	186 (36%)	141 (31%)	58 (26%)	
III	342 (28%)	131 (25%)	137 (30%)	74 (33%)	
IV	168 (14%)	60 (12%)	58 (12%)	50 (22%)	
MSI status					< 0.0001
MSI-low/MSS	1,071 (84%)	416 (75%)	433 (89%)	222 (93%)	
MSI-high	209 (16%)	141 (25%)	51 (11%)	17 (7.1%)	
*MLH1* hypermethylation					< 0.0001
Absent	1,073 (86%)	440 (79%)	413 (89%)	220 (94%)	
Present	179 (14%)	116 (21%)	50 (11%)	13 (5.6%)	
CIMP status					< 0.0001
Low/negative	1,032 (82%)	412 (74%)	403 (87%)	217 (93%)	
High	220 (18%)	144 (26%)	60 (13%)	16 (6.9%)	
*BRAF* mutation					< 0.0001
Wild-type	1,095 (85%)	440 (79%)	438 (89%)	217 (91%)	
Mutant	194 (15%)	120 (21%)	53 (11%)	21 (8.8%)	
*KRAS* mutation					0.88
Wild-type	729 (58%)	311 (59%)	281 (58%)	137 (58%)	
Mutant	519 (42%)	214 (41%)	204 (42%)	101 (42%)	
*PIK3CA* mutation					0.44
Wild-type	1,007 (84%)	443 (84%)	373 (83%)	191 (86%)	
Mutant	195 (16%)	86 (16%)	79 (17%)	30 (14%)	

CIMP, CpG island methylator phenotype; LINE-1, long interspersed nucleotide element-1; MSI, microsatellite instability; MSS, microsatellite stable; SD, standard deviation.

a Percentage (%) indicates the proportion of cases with a specific clinical, pathological, or tumor molecular feature in colorectal cancer cases with each tumor LINE-1 methylation level. There were cases that had missing values for any of the characteristics except for age, sex, year of diagnosis, and tumor location.

b To assess associations between categorical data, the chi-square test was performed. To compare mean age, an analysis of variance was performed. We adjusted two-sided α level to 0.003 (= 0.05/15) by simple Bonferroni correction for multiple hypothesis testing.

c TNM stage was based on the classification of the American Joint Committee on Cancer staging system.

Clinical, pathological, and tumor molecular features according to tumor LINE-1 methylation level in proximal colon, distal colon, and rectal cancers are summarized in Supplementary Table S1.

### Tumor LINE-1 hypomethylation and patient survival according to colorectal cancer location

We examined the relationship between tumor LINE-1 methylation level and patient survival according to colorectal cancer location (Table 2). In the 1,317 colorectal cancer cases, there were 717 deaths, including 382 colorectal cancer-specific deaths, during a median patient follow-up of 12.0 years (interquartile range: 8.0 to 16.6) among censored cases.

**Table 2:** Tumor LINE-1 hypomethylation and patient survival according to colorectal cancer location

	No. of cases	Colorectal cancer-specific mortality			Overall mortality		
		No. of events	Univariable HR (95% CI)	Multivariable HR (95% CI)^a^	No. of events	Univariable HR (95% CI)	Multivariable HR (95% CI)^a^
Proximal colon cancer							
LINE-1 hypomethylation (20% decrease as a unit)	621	176	2.37 (1.74-3.23)	1.66 (1.21- 2.28)	344	1.53 (1.21-1.93)	1.40 (1.10-1.78)
*P*^b^			< 0.0001	0.002		0.0004	0.007
Distal colon cancer							
LINE-1 hypomethylation (20% decrease as a unit)	409	111	1.30 (0.91-1.86)	1.18 (0.81- 1.72)	211	1.04 (0.79-1.36)	1.14 (0.86-1.50)
*P*^b^			0.16	0.40		0.78	0.36
Rectal cancer							
LINE-1 hypomethylation (20% decrease as a unit)	287	95	0.92 (0.60-1.41)	0.87 (0.57- 1.34)	162	0.79 (0.57-1.09)	0.72 (0.52-1.00)
*P*^b^			0.69	0.53		0.15	0.052
*P*_interaction_^c^			0.0002	0.011		0.0007	0.002

CI, confidence interval; HR, hazard ratio; LINE-1, long interspersed nucleotide element-1.

a The multivariable Cox regression model initially included sex, age, year of diagnosis, family history of colorectal cancer in parent or sibling, disease stage, tumor differentiation, microsatellite instability, CpG island methylator phenotype, and *KRAS*, *BRAF*, and *PIK3CA* mutations. A backward elimination with a threshold of *P* = 0.05 was used to select variables in the final models.

b *P* value was calculated by the Wald test (two-sided).

c *P*_interaction_ values (two-sided) were calculated by the Wald test on the cross-product term of tumor LINE-1 methylation level as a continuous variable and colorectal cancer location as an ordinal variable (proximal colon [1], distal colon [2], and rectum [3]).

**Figure 1:**
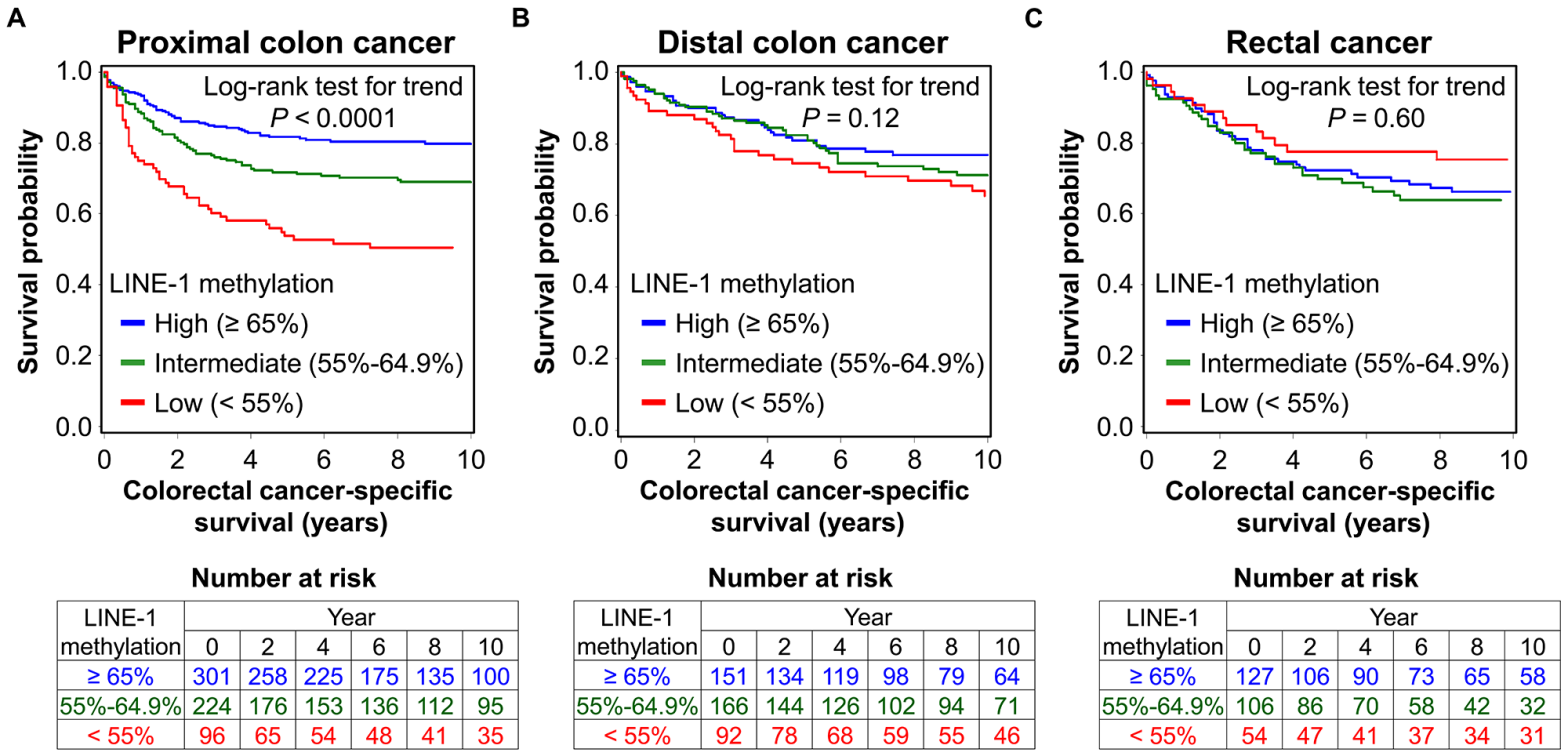
Kaplan-Meier curves for colorectal cancer-specific survival according to tumor LINE-1 methylation level in proximal colon cancer (A), distal colon cancer (B), and rectal cancer (C) *P* value was calculated by the log-rank test for trend (two-sided). The tables (bottom) show the number of patients who remained alive and at risk of death at each time point after the diagnosis of colorectal cancer.

For our primary hypothesis testing, we found a statistically significant interaction between LINE-1 methylation level and tumor location in colorectal cancer-specific mortality analysis (*P*_interaction_ = 0.011; Table 2). Hazard ratio (HR) of colorectal cancer-specific mortality for 20% decrease in tumor LINE-1 methylation level was higher in proximal colon cancers (multivariable HR, 1.66; 95% confidence interval [CI], 1.21 to 2.28) than in distal colon cancers (multivariable HR, 1.18; 95% CI, 0.81 to 1.72) or rectal cancers (multivariable HR, 0.87; 95% CI, 0.57 to 1.34) (Table 2). In the Kaplan-Meier analysis and the log-rank test (Figure 1), tumor LINE-1 hypomethylation was associated with higher colorectal cancer-specific mortality in proximal colon cancer (*P* < 0.0001 for trend), but not in distal colon cancer (*P* = 0.12 for trend) or rectal cancer (*P* = 0.60 for trend).

In the secondary analysis, a similar interactive association between tumor LINE-1 methylation level and colorectal cancer location was observed in overall mortality analysis (*P*_interaction_ = 0.002; Table 2). Tumor LINE-1 hypomethylation was associated with higher overall mortality in proximal colon cancer (for 20% decrease in tumor LINE-1 methylation level: multivariable HR, 1.40; 95% CI, 1.10 to 1.78), whereas tumor LINE-1 methylation level were not significantly associated with overall mortality in distal colon cancer (for 20% decrease in tumor LINE-1 methylation level: multivariable HR, 1.14; 95% CI, 0.86 to 1.50) or rectal cancer (for 20% decrease in tumor LINE-1 methylation level: multivariable HR, 0.72; 95% CI, 0.52 to 1.00).

### Tumor LINE-1 hypomethylation and patient survival in strata of colorectal tumor location and MSI status

Given our previous study showing the interactive association between tumor LINE-1 methylation level and MSI status in relation to colorectal cancer mortality [25], we conducted an exploratory analysis to examine the relationship between tumor LINE-1 hypomethylation and patient survival in strata of colorectal cancer location and MSI status (Table 3). In proximal colon cancers, tumor LINE-1 hypomethylation appeared to be associated with higher colorectal cancer-specific mortality not only in MSI-high cancers (for 20% decrease in tumor LINE-1 methylation level: multivariable HR, 6.14; 95% CI, 2.27 to 16.6) but also in MSI-low/MSS cancers (for 20% decrease in tumor LINE-1 methylation level: multivariable HR, 1.44; 95% CI, 1.04 to 2.01).

**Table 3:** Tumor LINE-1 hypomethylation and patient survival according to colorectal cancer location and MSI status

	No. of cases	Colorectal cancer-specific mortality			Overall mortality		
		No. of events	Univariable HR (95% CI)	Multivariable HR (95% CI)^a^	No. of events	Univariable HR (95% CI)	Multivariable HR (95% CI)^a^
MSI-low/MSS proximal colon cancer							
LINE-1 hypomethylation (20% decrease as a unit)	440	153	1.77 (1.26-2.50)	1.44 (1.04-2.01)	259	1.41 (1.07-1.84)	1.27 (0.97-1.66)
*P*^b^			0.001	0.030		0.014	0.08
MSI-high proximal colon cancer							
LINE-1 hypomethylation (20% decrease as a unit)	181	23	4.51 (1.78-11.4)	6.14 (2.27-16.6)	85	1.62 (0.96-2.74)	2.30 (1.30-4.06)
*P*^b^			0.002	0.0003		0.07	0.004
MSI-low/MSS distal colon cancer							
LINE-1 hypomethylation (20% decrease as a unit)	388	108	1.21 (0.84-1.76)	1.13 (0.76-1.66)	203	0.97 (0.73-1.28)	1.06 (0.80-1.41)
*P*^b^			0.31	0.55		0.82	0.69
MSI-high distal colon cancer							
LINE-1 hypomethylation (20% decrease as a unit)	21	3	3.05 (0.74-12.6)	2.76 (0.75-10.1)	8	2.38 (0.89-6.36)	2.00 (0.87-4.60)
*P*^b^			0.12	0.13		0.08	0.10
Rectal cancer							
LINE-1 hypomethylation (20% decrease as a unit)	287	95	0.92 (0.60-1.40)	0.87 (0.57-1.34)	162	0.79 (0.57-1.09)	0.72 (0.52-1.00)
*P*^b^			0.69	0.52		0.15	0.052

CI, confidence interval; HR, hazard ratio; LINE-1, long interspersed nucleotide element-1; MSI, microsatellite instability; MSS, microsatellite stable.

a The multivariable Cox regression model initially included sex, age, year of diagnosis, family history of colorectal cancer in parent or sibling, disease stage, tumor differentiation, CpG island methylator phenotype, and *KRAS*, *BRAF*, and *PIK3CA* mutations. A backward elimination with a threshold of *P* = 0.05 was used to select variables in the final models.

b *P* value was calculated by the Wald test (two-sided).

## DISCUSSION

We conducted this study to test the hypothesis that the prognostic association of tumor LINE-1 hypomethylation might differ by colorectal cancer location. Utilizing the database of 1,317 colorectal cancer cases in the two U.S.-nationwide prospective cohort studies, we found a statistically significant interactive association of tumor LINE-1 methylation level and tumor location in colorectal cancer mortality analysis. The adverse prognostic association of tumor LINE-1 hypomethylation was stronger in proximal colon cancers than in distal colorectal cancers.

Colorectal cancers are a heterogeneous group of diseases that result from the accumulation of differing sets of genomic and epigenomic alterations, and tumor-host interactions and, hence, research on tumor biomarkers is important for clinical medicine and public health [26–32]. Few studies have considered colorectal cancer location in evaluating clinical outcome by tumor LINE-1 methylation level. In 76 patients with stage III proximal colon cancer, a lower tumor LINE-1 methylation level is associated with poor disease-free survival [23]. In 94 patients with stage I and II rectal cancer, a lower tumor LINE-1 methylation level is associated with poor recurrence-free and overall survival [33]. However, these previous studies were limited by small sample sizes, and did not examine the interactive association of tumor LINE-1 methylation level and tumor location with clinical outcome. By utilizing a large collection (n = 1,317) of colorectal cancer cases, our population-based data support the interactive association of tumor LINE-1 methylation level and tumor location in colorectal cancer mortality analysis.

Exact mechanisms by which colorectal tumors that exhibit LINE-1 hypomethylation have been associated with aggressive tumor behavior remain uncertain. It has been known that colorectal cancers develop through the accumulation of genetic and epigenetic alterations, influenced by microbial and other environmental exposures and host responses to the exposures [34–39]. Our previous study has shown that the frequencies of key molecular features such as MSI, CIMP-high, *BRAF* and *PIK3CA* mutations change gradually along the length of the colorectum [7], suggesting effects of gut contents and microbiota on colorectal tumorigenesis [40]. It is conceivable that tumor-host interactions in the tumor microenvironment including immune response and inflammation might influence the progression of colorectal tumors that exhibit LINE-1 hypomethylation. An increasing body of evidence suggests that microbiota can influence host immunity, and that microbiota, luminal contents, and colonic mucosal immunity may change from the proximal to distal colorectal segments [5, 6, 12, 41–44], future investigations are necessary to examine effects of microbiota on colorectal tumor progression.

Studies have shown that tumor LINE-1 hypomethylation in colorectal cancer is inversely associated with MSI-high, and that the prognostic association of tumor LINE-1 hypomethylation in colorectal cancer is stronger in MSI-high colorectal cancers than in MSS colorectal cancers [24, 25, 45–47], suggesting a complex biological interaction between MSI and LINE-1 hypomethylation in colorectal tumor progression. MSI-high colorectal cancers have been characterized by numerous somatic mutations [48], which might interact with genomic DNA methylation. Future studies are needed to clarify the underlying mechanisms of the association between tumor LINE-1 methylation and MSI status in colorectal cancer progression.

There are biological and clinical differences between colon and rectal cancers including treatment approaches and metastatic pattern [49]. Multiple studies have demonstrated that proportions of colorectal cancers with specific molecular features such as MSI, high-level CIMP, and *BRAF* and *PIK3CA* mutations gradually increase along the bowel subsites from rectum to ascending colon [7–9]. Hence, we included both colon and rectal carcinoma cases in the current study. This continuum model is a more advanced model than the simple colon-vs-rectum dichotomy model, because a continuous difference (along the colorectum) will surely lead to a difference in the dichotomy model. We have examined proximal colon vs. distal colon vs. rectum, considering statistical power. Future studies with larger sample size are needed to investigate the prognostic significance of tumor molecular features in the detailed colorectal cancer subsites.

Previous studies have shown that tumor LINE-1 hypomethylation in colorectal cancer is associated with worse clinical outcome, and that LINE-1 hypomethylated colorectal cancers are associated with young age of onset and a family history of colorectal cancer [21, 47, 50]. These findings suggest that LINE-1 methylation level may serve as a potential tumor biomarker for prognosis as well as for familial cancer risk assessment. In addition, our current study found that the adverse prognostic association of tumor LINE-1 hypomethylation was stronger in proximal colon cancers than in distal colorectal cancers. Our current data may help us further stratify patients with colorectal cancer into more individualized prognostic groups based on tumor location, and can guide future mechanistic studies.

We acknowledge limitations of our current study. First, data on cancer recurrence were limited in the two cohorts and were not examined. However, colorectal cancer-specific mortality is a reasonable cancer-specific outcome in the current study, which utilized the population-based data of long-term patient follow-up, since median survival for recurrent (local or metastatic) colorectal cancer was approximately 10 to 20 months during much of the time period of this study [51]. Second, the information on cancer treatment including chemotherapy use and regimens was also limited. However, distributions of chemotherapy use and its regimens would unlikely substantially differ according to tumor LINE-1 methylation level, because these data were not generally utilized for treatment decisions. The comprehensive genome-scale DNA methylation analysis in colorectal cancer by Hinoue et al. [52] has suggested distinct DNA methylation subgroups of colorectal cancer. Although we assessed well-established LINE-1 methylation as a surrogate of global DNA methylation status, this method might not capture methylation status in other CpG sites. Despite this limitation, LINE-1 hypomethylation was significantly associated with mortality in proximal colon cancer patients. This finding suggests the importance and clinical usefulness of LINE-1 methylation status, while more comprehensive methylation profile is warranted to gain new insights into roles of DNA methylation in carcinogenesis.

Strengths of this study include the use of the molecular pathological epidemiology [53–56] database of 1,317 colon and rectal carcinoma cases within the two U.S.-nationwide prospective cohort studies. Importantly, our colorectal cancer sample represented a group of patients in a large number of hospitals in diverse settings across the U.S., which increases the generalizability of our findings. Additionally, our database integrates clinical, pathologic, and key tumor molecular features of colorectal cancer. Finally, the sample size and the comprehensiveness of this population-based colorectal cancer database enabled us to achieve a sufficient statistical power to assess the interactive association of tumor LINE-1 methylation level and colorectal cancer location with colorectal cancer mortality, controlling for potential confounders.

In conclusion, we have shown that the adverse prognostic association of tumor LINE-1 hypomethylation is stronger in proximal colon cancers than in distal colorectal cancers. Our data suggest the interactive effect of tumor LINE-1 methylation level and tumor location on clinical outcome.

## MATERIALS AND METHODS

### Study population

We utilized the database of colon and rectal carcinoma cases in two U.S.-nationwide prospective cohort studies, the NHS (121,701 women enrolled in 1976) and the HPFS (51,529 men enrolled in 1986) [57, 58]. Every 2 years, we sent follow-up questionnaires to collect information on lifestyle factors, and asked whether they had received diagnoses of major disease including cancers. The National Death Index was used to identify unreported fatal colorectal cancer cases. Study physicians reviewed medical records for colorectal cancer cases, and assigned the cause of death for all deceased cases. We collected formalin-fixed paraffin-embedded tissue blocks from hospitals across the United States where participants with colorectal cancer had undergone tumor resection. A single pathologist (S.O.), who was unaware of other data, reviewed hematoxylin and eosin-stained tissue sections of all colorectal carcinoma cases, and recorded pathological features. Tumor differentiation was classified as well to moderate vs. poor (>50% vs. ≤50% glandular area). We analyzed available data on tumor LINE-1 methylation level, tumor location, and patient survival among 1,317 colorectal cancer cases diagnosed up to 2008. Patients were followed until death or January 1, 2012, whichever came first. The procedures and protocols of this study were approved by the institutional review boards for the Harvard T.H. Chan School of Public Health and the Brigham and Women's Hospital (Boston, MA, USA).

### Assessment of tumor location

Study physicians, unaware of other data, reviewed medical and pathological reports, and recorded tumor location (cecum, ascending colon, hepatic flexure, transverse colon, splenic flexure, descending colon, sigmoid colon, rectosigmoid, and rectum). Proximal colon consists of cecum, ascending colon, hepatic flexure, and transverse colon, whereas distal colon consists of splenic flexure, descending colon, and sigmoid colon.

### Analysis of LINE-1 methylation level

DNA was extracted from archival colorectal cancer tissue blocks. We performed bisulfite treatment of DNA, polymerase chain reaction (PCR), and a pyrosequencing assay to measure tumor LINE-1 methylation levels after assay validation [59]. We primarily used tumor LINE-1 methylation level as a continuous variable (20% decrease as a unit) in survival analyses. When we displayed tumor LINE-1 methylation level in relation to clinical, pathological, and tumor molecular features (Table 1), we categorized tumor LINE-1 methylation levels into low (<55% methylation) vs. intermediate (55-64.9% methylation) vs. high (≥65% methylation) to keep consistency with our previous studies [25, 50].

### Analyses of MSI, CIMP, and *KRAS*, *BRAF*, and *PIK3CA* mutations

MSI status was analyzed with use of 10 microsatellite markers (D2S123, D5S346, D17S250, BAT25, BAT26, BAT40, D18S55, D18S56, D18S67, and D18S487) as previously described [60]. We defined MSI-high as the presence of instability in ≥30% of the markers, and MSI-low/microsatellite stable (MSS) as instability in <30% of the markers. Methylation analysis of eight promoter CpG islands specific for CIMP (*CACNA1G*, *CDKN2A*, *CRABP1*, *IGF2*, *MLH1*, *NEUROG1*, *RUNX3*, and *SOCS1*) was performed as previously described [61, 62]. PCR and pyrosequencing assay targeted for *KRAS* (codons 12, 13, 61, and 146) [63, 64], *BRAF* (codon 600) [60], and *PIK3CA* (exons 9 and 20) [65, 66] were performed as previously described.

### Statistical analysis

All statistical analyses were conducted using SAS (version 9.3, SAS Institute, Cary, NC) and all *P* values were two-sided. Our primary hypothesis testing was a statistical interaction of tumor LINE-1 methylation level and colorectal cancer location in colorectal cancer-specific mortality analysis. Overall mortality was a secondary outcome. The statistical interaction was assessed by the Wald test on the cross-product term of tumor LINE-1 methylation level as a continuous variable and colorectal cancer location as an ordinal variable (proximal colon [1], distal colon [2], and rectum [3]) in a Cox proportional hazards regression model. A two-sided α level was set at 0.05 for our primary hypothesis testing. For a secondary or exploratory analysis, we adjusted the two-sided α level by simple Bonferroni correction for multiple hypothesis testing, in addition to the use of the two-sided α level of 0.05 for our primary hypothesis testing.

For analyses of colorectal cancer-specific mortality, deaths as a result of other causes were censored. The Kaplan-Meier method was used to describe the distribution of colorectal cancer-specific survival, and the log-rank test for trend was performed to assess a linear trend in survival probability across the ordinal categories (high [1] vs. intermediate [2] vs. low [3]) of tumor LINE-1 methylation level. To control for confounders, we used multivariable Cox proportional hazards regression models. In addition to the tumor LINE-1 hypomethylation variable (continuous; 20% decrease as a unit), the multivariable model initially included sex, age at diagnosis (continuous), year of diagnosis (continuous), family history of colorectal cancer in a first-degree relative (present vs. absent), disease stage (I/II vs. III/IV), tumor differentiation (well to moderate vs. poor), MSI (high vs. MSI-low/MSS), CIMP (high vs. low/negative), *KRAS* (mutant vs. wild-type), *BRAF* (mutant vs. wild-type), and *PIK3CA* (mutant vs. wild-type). A single analysis model could assess the prognostic association of tumor LINE-1 hypomethylation in each stratum of colorectal cancer location, using a reparameterization of the interaction term (of tumor LINE-1 hypomethylation and colorectal cancer location) as previously described [25, 57]. A backward elimination was carried out with *P* = 0.05 as a threshold, to select variables for the final model. For cases with missing information in any of the categorical covariates (family history of colorectal cancer in a first-degree relative [0.4%], disease stage [8.7%], tumor differentiation [0.6%], MSI [2.8%], CIMP [4.9%], *KRAS* [5.2%], *BRAF* [2.1%], and *PIK3CA* [8.7%]), we included these cases in the majority category of a given covariate to minimize the number of variables in the multivariable Cox models. We confirmed that excluding the cases with missing information in any of the covariates did not substantially alter results (data not shown). The proportionality of hazards assumption was assessed by a time-varying covariate, using an interaction term of colorectal cancer-specific survival and tumor LINE-1 methylation level (*P* = 0.97).

All univariable analyses for associations of tumor LINE-1 methylation level with clinical, pathological, and tumor molecular features in colorectal cancer, and these associations according to proximal colon, distal colon, and rectal cancer were considered as secondary exploratory analyses. Given the 13 covariates in overall colorectal cancer and the 12 covariates in each of the three cancer locations, we adjusted the two-sided α level to 0.003 (= 0.05/15) by simple Bonferroni correction for multiple hypothesis testing. To assess associations between categorical variables, the chi-square test was performed. To compare mean age, an analysis of variance assuming equal variances was performed.

### Abbreviations

CI, confidence interval; CIMP, CpG island methylator phenotype; HPFS, Health Professionals Follow-up Study; HR, hazard ratio; LINE-1, long interspersed nucleotide element-1; MSI, microsatellite instability; MSS, microsatellite stable; NHS, Nurses’ Health Study; PCR, polymerase chain reaction; SD, standard deviation.

### Use of standardized official symbols

We use HUGO (Human Genome Organisation)-approved official symbols for genes and gene products, including BRAF, CACNA1G, CDKN2A, CRABP1, IGF2, KRAS, MLH1, NEUROG1, PIK3CA, RUNX3, and SOCS1; all of which are described at www.genenames.org. Gene names are italicized, and gene product names are non-italicized.

## SUPPLEMENTARY TABLE



## Abbreviations

CI: confidence interval
CIMP: CpG island methylator phenotype
HPFS: Health Professionals Follow-up Study
HR: hazard ratio
LINE-1: long interspersed nucleotide element-1
MSI: microsatellite instability
MSS: microsatellite stable
NHS: Nurses’ Health Study
PCR: polymerase chain reaction
SD: standard deviation.
