# Supplementary material for: Tumor LINE-1 methylation level and colorectal cancer location in relation to patient survival
Source: Oncotarget. 2016 Jul 4;7(34):55098–109. doi: 10.18632/oncotarget.10398 (PMC5342404; doi:10.18632/oncotarget.10398)
Supplement: Supplementary file 1 [file oncotarget-07-55098-s001.doc]

Supplementary Table S1. Clinical, pathological, and tumor molecular features according to tumor LINE-1 methylation level in proximal colon, distal colon, and rectal cancer

|  | Proximal colon cancer (n = 621) | | | |  | Distal colon cancer (n = 409) | | | |  | Rectal cancer (n = 287) | | | |
| --- | --- | --- | --- | --- | --- | --- | --- | --- | --- | --- | --- | --- | --- | --- |
|  | Tumor LINE-1 methylation level | | |  |  | Tumor LINE-1 methylation level | | |  |  | Tumor LINE-1 methylation level | | |  |
| Characteristic^a^ | ≥65%  (n =301) | 55-64.9%  (n = 224) | <55%  (n = 96) | *P* value^b^ |  | ≥65%  (n = 151) | 55-64.9%  (n = 166) | <55%  (n = 92) | *P* value^b^ |  | ≥65%  (n = 127) | 55-64.9%  (n = 106) | <55%  (n = 54) | *P* value^b^ |
| Mean tumor LINE-1 methylation level  (%) ± SD | 72.4 ± 5.5 | 60.9 ± 2.7 | 49.2 ± 5.7 |  |  | 72.1 ± 5.9 | 60.2 ± 2.9 | 48.3 ± 6.5 |  |  | 71.1 ± 4.8 | 60.2 ± 2.6 | 49.5 ± 5.4 |  |
|  |  |  |  |  |  |  |  |  |  |  |  |  |  |  |
| Mean age ± SD (year) | 71.1 ± 8.4 | 69.6 ± 8.3 | 68.5 ± 8.8 | 0.015 |  | 69.2 ± 8.7 | 67.0 ± 8.2 | 65.9 ± 8.8 | 0.008 |  | 68.0 ± 8.6 | 69.3 ± 9.8 | 66.8 ± 10.0 | 0.25 |
|  |  |  |  |  |  |  |  |  |  |  |  |  |  |  |
| Sex |  |  |  | 0.94 |  |  |  |  | 0.037 |  |  |  |  | 0.50 |
| Men | 123 (41%) | 95 (42%) | 40 (42%) |  |  | 70 (46%) | 68 (41%) | 53 (58%) |  |  | 62 (49%) | 51 (48%) | 31 (57%) |  |
| Women | 178 (59%) | 129 (58%) | 56 (58%) |  |  | 81 (54%) | 98 (59%) | 39 (42%) |  |  | 65 (51%) | 55 (52%) | 23 (43%) |  |
|  |  |  |  |  |  |  |  |  |  |  |  |  |  |  |
| Year of diagnosis |  |  |  | < 0.0001 |  |  |  |  | < 0.0001 |  |  |  |  | 0.39 |
| Prior to 1995 | 67 (22%) | 84 (38%) | 40 (42%) |  |  | 45 (30%) | 83 (50%) | 42 (46%) |  |  | 50 (39%) | 35 (33%) | 22 (41%) |  |
| 1996 to 2000 | 77 (26%) | 79 (35%) | 40 (42%) |  |  | 41 (27%) | 38 (23%) | 39 (42%) |  |  | 39 (31%) | 30 (28%) | 19 (35%) |  |
| 2001 to 2008 | 157 (52%) | 61 (27%) | 16 (16%) |  |  | 65 (43%) | 45 (27%) | 11 (12%) |  |  | 38 (30%) | 41 (39%) | 13 (24%) |  |
|  |  |  |  |  |  |  |  |  |  |  |  |  |  |  |
| Family history of colorectal cancer in a first-degree relative | |  |  | 0.87 |  |  |  |  | 0.39 |  |  |  |  | 0.19 |
| Absent | 232 (77%) | 170 (76%) | 71 (75%) |  |  | 126 (83%) | 128 (78%) | 72 (78%) |  |  | 113 (89%) | 93 (89%) | 43 (80%) |  |
| Present | 68 (23%) | 53 (24%) | 24 (25%) |  |  | 25 (17%) | 37 (22%) | 20 (22%) |  |  | 14 (11%) | 12 (11%) | 11 (20%) |  |
|  |  |  |  |  |  |  |  |  |  |  |  |  |  |  |
| Tumor differentiation | |  |  | 0.06 |  |  |  |  | 0.015 |  |  |  |  | 0.28 |
| Well to moderate | 243 (81%) | 197 (88%) | 77 (80%) |  |  | 139 (92%) | 163 (99%) | 86 (93%) |  |  | 119 (95%) | 101 (96%) | 53 (100%) |  |
| Poor | 56 (19%) | 26 (12%) | 19 (20%) |  |  | 12 (8.0%) | 2 (1.2%) | 6 (6.5%) |  |  | 6 (4.8%) | 4 (3.8%) | 0 |  |
|  |  |  |  |  |  |  |  |  |  |  |  |  |  |  |
| pT stage (depth of tumour invasion) |  |  |  | 0.26 |  |  |  |  | 0.73 |  |  |  |  | 0.84 |
| pT1 (submucosa) | 22 (7.9%) | 13 (6.1%) | 2 (2.2%) |  |  | 23 (17%) | 32 (21%) | 9 (11%) |  |  | 17 (15%) | 18 (18%) | 7 (14%) |  |
| pT2 (muscularis propria) | 59 (21%) | 40 (19%) | 15 (17%) |  |  | 23 (17%) | 26 (17%) | 16 (20%) |  |  | 33 (29%) | 24 (24%) | 13 (26%) |  |
| pT3 (subserosa) | 183 (66%) | 144 (68%) | 63 (70%) |  |  | 86 (63%) | 89 (58%) | 51 (65%) |  |  | 58 (51%) | 55 (56%) | 28 (56%) |  |
| pT4 (serosa or other organs) | 15 (5.4%) | 15 (7.1%) | 10 (11%) |  |  | 5 (3.7%) | 6 (3.9%) | 3 (3.8%) |  |  | 6 (5.3%) | 2 (2.0%) | 2 (4.0%) |  |
|  |  |  |  |  |  |  |  |  |  |  |  |  |  |  |
| pN stage (number of positive lymph nodes) |  |  |  | 0.009 |  |  |  |  | 0.32 |  |  |  |  | 0.77 |
| pN0 (0) | 199 (72%) | 133 (63%) | 45 (54%) |  |  | 88 (68%) | 93 (63%) | 42 (54%) |  |  | 61 (57%) | 54 (59%) | 25 (51%) |  |
| pN1 (1-3) | 51 (19%) | 45 (22%) | 20 (24%) |  |  | 28 (21%) | 40 (27%) | 22 (29%) |  |  | 29 (27%) | 20 (22%) | 13 (27%) |  |
| pN2 (≥4) | 25 (9.1%) | 32 (15%) | 18 (22%) |  |  | 14 (11%) | 15 (10%) | 13 (17%) |  |  | 17 (16%) | 18 (19%) | 11 (22%) |  |
|  |  |  |  |  |  |  |  |  |  |  |  |  |  |  |
| TNM stage^c^ |  |  |  | < 0.0001 |  |  |  |  | 0.21 |  |  |  |  | 0.58 |
| I | 70 (25%) | 46 (22%) | 13 (14%) |  |  | 34 (26%) | 44 (29%) | 17 (20%) |  |  | 37 (35%) | 34 (36%) | 13 (26%) |  |
| II | 117 (42%) | 80 (37%) | 25 (27%) |  |  | 50 (37%) | 42 (28%) | 22 (27%) |  |  | 19 (18%) | 19 (20%) | 11 (22%) |  |
| III | 57 (21%) | 61 (28%) | 24 (26%) |  |  | 31 (23%) | 46 (31%) | 29 (35%) |  |  | 43 (41%) | 30 (31%) | 21 (42%) |  |
| IV | 34 (12%) | 28 (13%) | 30 (33%) |  |  | 19 (14%) | 18 (12%) | 15 (18%) |  |  | 7 (6.6%) | 12 (13%) | 5 (10%) |  |
|  |  |  |  |  |  |  |  |  |  |  |  |  |  |  |
| MSI status |  |  |  | < 0.0001 |  |  |  |  | 0.003 |  |  |  |  | 0.91 |
| MSI-low/MSS | 167 (58%) | 175 (80%) | 82 (86%) |  |  | 131 (90%) | 161 (98%) | 87 (97%) |  |  | 118 (98%) | 97 (97%) | 53 (98%) |  |
| MSI-high | 123 (42%) | 45 (20%) | 13 (14%) |  |  | 15 (10%) | 3 (1.8%) | 3 (3.3%) |  |  | 3 (2.5%) | 3 (3.0%) | 1 (1.9%) |  |
|  |  |  |  |  |  |  |  |  |  |  |  |  |  |  |
| *MLH1* hypermethylation |  |  |  | < 0.0001 |  |  |  |  | 0.014 |  |  |  |  | 0.21 |
| Absent | 186 (65%) | 165 (78%) | 82 (90%) |  |  | 134 (92%) | 154 (98%) | 88 (98%) |  |  | 120 (98%) | 94 (100%) | 50 (96%) |  |
| Present | 101 (35%) | 47 (22%) | 9 (9.9%) |  |  | 12 (8.2%) | 3 (1.9%) | 2 (2.2%) |  |  | 3 (2.4%) | 0 | 2 (3.9%) |  |
|  |  |  |  |  |  |  |  |  |  |  |  |  |  |  |
| CIMP status |  |  |  | < 0.0001 |  |  |  |  | 0.0008 |  |  |  |  | 0.41 |
| Low/negative | 164 (57%) | 157 (74%) | 78 (86%) |  |  | 129 (88%) | 154 (98%) | 87 (97%) |  |  | 119 (97%) | 92 (98%) | 52 (100%) |  |
| High | 123 (43%) | 55 (26%) | 13 (14%) |  |  | 17 (12%) | 3 (1.9%) | 3 (3.3%) |  |  | 4 (3.3%) | 2 (2.1%) | 0 |  |
|  |  |  |  |  |  |  |  |  |  |  |  |  |  |  |
| *BRAF* mutation |  |  |  | < 0.0001 |  |  |  |  | 0.021 |  |  |  |  | 0.76 |
| Wild-type | 191 (66%) | 174 (79%) | 81 (85%) |  |  | 131 (89%) | 161 (97%) | 83 (93%) |  |  | 118 (97%) | 103 (98%) | 53 (98%) |  |
| Mutant | 100 (34%) | 46 (21%) | 14 (15%) |  |  | 16 (11%) | 5 (3.0%) | 6 (6.7%) |  |  | 4 (3.3%) | 2 (1.9%) | 1 (1.9%) |  |
|  |  |  |  |  |  |  |  |  |  |  |  |  |  |  |
| *KRAS* mutation |  |  |  | 0.27 |  |  |  |  | 0.24 |  |  |  |  | 0.31 |
| Wild-type | 163 (60%) | 120 (55%) | 49 (52%) |  |  | 76 (57%) | 94 (58%) | 60 (67%) |  |  | 72 (60%) | 67 (64%) | 28 (52%) |  |
| Mutant | 108 (40%) | 99 (45%) | 46 (48%) |  |  | 58 (43%) | 68 (42%) | 29 (33%) |  |  | 48 (40%) | 37 (36%) | 26 (48%) |  |
|  |  |  |  |  |  |  |  |  |  |  |  |  |  |  |
| *PIK3CA* mutation |  |  |  | 0.11 |  |  |  |  | 0.63 |  |  |  |  | 0.50 |
| Wild-type | 232 (84%) | 155 (77%) | 73 (85%) |  |  | 111 (80%) | 129 (84%) | 70 (84%) |  |  | 100 (87%) | 89 (91%) | 48 (92%) |  |
| Mutant | 44 (16%) | 46 (23%) | 13 (15%) |  |  | 27 (20%) | 24 (16%) | 13 (16%) |  |  | 15 (13%) | 9 (9.2%) | 4 (7.7%) |  |

CIMP, CpG island methylator phenotype; LINE-1, long interspersed nucleotide element-1, MSI, microsatellite instability; MSS, microsatellite stable; SD, standard deviation.

^a^ Percentage (%) indicates the proportion of cases with a specific clinical, pathological, or tumor molecular feature in proximal colon, distal colon, and rectal cancer cases with each tumor LINE-1 methylation level. There were cases that had missing values for any of the characteristics except for age, sex, and year of diagnosis.

^b^ To assess associations between categorical variables, the chi-square test was performed. To compare mean age, an analysis of variance was performed.

We adjusted two-sided α level to 0.004 by simple Bonferroni correction for multiple hypothesis testing.

^c^ TNM stage was based on the classification of the American Joint Committee on Cancer staging system.
